# Building Kidney Exchange Programmes in Europe—An Overview of Exchange Practice and Activities

**DOI:** 10.1097/TP.0000000000002432

**Published:** 2019-06-26

**Authors:** Péter Biró, Bernadette Haase-Kromwijk, Tommy Andersson, Eyjólfur Ingi Ásgeirsson, Tatiana Baltesová, Ioannis Boletis, Catarina Bolotinha, Gregor Bond, Georg Böhmig, Lisa Burnapp, Katarína Cechlárová, Paola Di Ciaccio, Jiri Fronek, Karine Hadaya, Aline Hemke, Christian Jacquelinet, Rachel Johnson, Rafal Kieszek, Dirk R. Kuypers, Ruthanne Leishman, Marie-Alice Macher, David Manlove, Georgia Menoudakou, Mikko Salonen, Bart Smeulders, Vito Sparacino, Frits C.R. Spieksma, María Oliva Valentín, Nic Wilson, Joris van der Klundert

**Affiliations:** 1 Institute of Economics, Hungarian Academy of Sciences, Budapest, Hungary.; 2 Dutch Transplantation Foundation, Leiden, the Netherlands.; 3 Department of Economics, Lund University, Lund, Sweden.; 4 School of Science and Engineering, Reykjavik University, Reykjavik, Iceland.; 5 Transplant Center, L. Pasteur’s University Hospital, Košice, Slovakia.; 6 Nephrology Department, Laikon Hospital, Athens, Greece.; 7 Instituto Portugues do Sangue e da Transplantacao, Lisbon, Portugal.; 8 Division of Nephrology and Dialysis, Department of Medicine III, Medical University of Vienna, Vienna, Austria.; 9 National Health Service Blood and Transplant, Bristol, United Kingdom.; 10 Institute of Mathematics, P.J. Safarik University, Košice, Slovakia.; 11 Italian National Transplant Centre, Rome, Italy.; 12 Institute for Clinical and Experimental Medicine, Prague, Czech Republic.; 13 Division of Nephrology and Transplantation, Geneva University Hospitals, Geneva, Switzerland.; 14 Agence de la Biomedecine, Saint-Denis, France.; 15 Medical University of Warsaw, Warsaw, Poland.; 16 Department of Nephrology and Renal Transplantation, University Hospitals Leuven, Belgium.; 17 United Network for Organ Sharing (UNOS), Richmond. VA.; 18 School of Computing Sciences, University of Glasgow, Glasgow, United Kingdom.; 19 Hellenic Transplant Organization, Athens, Greece.; 20 Department of Political and Economic Studies, University of Helsinki, Helsinki, Finland.; 21 HEC Management School, Université de Liege, Liege, Belgium.; 22 Organización Nacional de Trasplantes, Madrid, Spain.; 23 Department of Computer Science, University College Cork, Cork, Ireland.; 24 Prince Mohammad Bin Salman College, King Abdullah Economic City, Kingdom of Saudi Arabia.

## Abstract

**Background.:**

Considerable differences exist among the living donor Kidney Exchange Programmes (KEPs) that are in use and being built in Europe, contributing to a variation in the number of living donor transplants (Newsletter Transplant; International figures on donation and transplantation 2016). Efforts of European KEPs to exchange (best) practices and share approaches to address challenges have, however, been limited.

**Methods.:**

Experts from 23 European countries, collaborating on the European Network for Collaboration on Kidney Exchange Programmes Cooperation on Science and Technology Action, developed a questionnaire to collect detailed information on the functioning of all existing KEPs in Europe, as well as their opportunities and challenges. Following a comparative analysis, results were synthesized and interpreted by the same experts.

**Results.:**

The practices, opportunities and challenges reported by 17 European countries reveal that some of the 10 operating programs are mature, whereas others are in earlier stages of development. Over 1300 transplants were performed through existing KEPs up to the end of 2016, providing approximately 8% of their countries’ living kidney donations in 2015. All countries report challenges to either initiating KEPs or increasing volumes. Some challenges are shared, whereas others differ because of differences in context (eg, country size, effectiveness of deceased donor program) and ethical and legal considerations (eg, regarding living donation as such, nonrelated donors, and altruistic donation). Transnational initiatives have started in Central Europe, Scandinavia, and Southern Europe.

**Conclusions.:**

Exchange of best practices and shared advancement of national programs to address existing challenges, aided by transnational exchanges, may substantially improve access to the most (cost) effective treatment for the increasing number of patients suffering from kidney disease.

For patients suffering from end-stage renal disease (ESRD), living donor kidney transplantation (LDKT) is more (cost) effective than deceased donor transplantation and dialysis.^1-3^ In particular, it yields better patient and graft survival.^4^ Over the past 2 decades, many countries have developed or intensified LDKT programs, often to supplement shortages in supply of donor organs for deceased donor programs. Other countries however, still largely rely on deceased donor programs. As of 2015, LDKT accumulates to 40% of approximately 80000 kidney transplantations worldwide.^5^

The differences in adoption and implementation of LDKT programs in Europe result in a variation from 0 to 33.2 in the number of living donor transplants per million inhabitants per year.^6^ This variation indicates that initiating and advancing KEPs can bring the benefit of treating more European ESRD patients, and/or treating them more (cost)-effectively.

Originally, LDKT was only feasible for patients with intended living donors of compatible blood group and HLA type. Depending on the country, 40% or more of recipients are incompatible with their intended donors.^5^ Kidney Exchange Programmes (KEPs) enable transplantation for recipients who are blood- and/or HLA incompatible with their initially intended donors. These incompatible pairs can join a larger pool of recipient-donor pairs, within which recipients may exchange donors. The KEP management identifies matching donors for the recipients and arranges the transplantations to take place. Many countries across the globe, and specifically in Europe, are establishing KEPs and facing a variety of common challenges.^7^ Additionally, some of the countries with existing KEPs appear to face challenges in increasing transplant numbers, despite considerable demand. Hence, there appears to be much to gain from collaboration and mutual learning.

The Cooperation on Science and Technology Association funds such a collaboration and mutual-learning project, namely the European Network for Collaboration on Kidney Exchange Programmes (ENCKEP) Cooperation on Science and Technology (COST) Action.^8^ The COST Association is a European framework whose actions are open to all 36 member-states, which is a wider set of countries than the EU and includes, eg, Norway and Turkey.^9^ In addition, participation is possible from Cooperating State Israel, Near Neighbor Countries, and International Partner Countries. Hence, we have used a wider definition of Europe in this article, allowing also collaboration with and mutual learning from non-European countries.

ENCKEP started on September 1, 2016, bringing together policy makers, clinicians, and optimization experts from 27 participating countries at the time of writing. The aims of ENCKEP are to (1) Exchange best practices and scientific state of the art with respect to national KEPs; (2) Develop a jointly used common framework for data and optimization; (3) Develop and test a prototype for transnational KEPs; (4) Stimulate European policy dialogue on this topic. In this study, we report on research conducted to achieve the first aforementioned aim. We provide a comparative analysis of Europe’s current KEP practices and challenges.

## MATERIALS AND METHODS

To systematically describe current KEP practices in Europe, a questionnaire has been developed to identify similarities and differences between countries, and future challenges. The questionnaire did not include individual patient or donor data and therefore no ethics board approval was needed.

The questionnaire was based on an earlier questionnaire that had been designed for the European Committee on Organ Transplantation within the Council of Europe (CD-P-TO).^10^ This original questionnaire was reviewed, amended, and restructured, following expert panel discussion and involving representatives from the participating countries. These discussions were conducted partly at the first workshop of the COST Action in January 2017,^8^ where most of the operating European KEPs were presented, and their main features were identified. The detailed written reports of the discussion were subsequently restructured into a new questionnaire by a smaller expert group, and then distributed for improvement suggestions to ENCKEP participants, resulting in a final commonly approved questionnaire. The questionnaire was divided into sections focusing on:

–Short country description
–Numbers of registration and transplantation
–Activities
–Challenges and opportunities.

The questionnaires were sent to representatives of the ENCKEP countries in February 2017, who were asked to address all questions applicable to their country by March 2017. The data collected through the questionnaires were systematically presented and subsequently synthesized into conclusions and recommendations in a workshop in March 2017,^8^ by representatives of the participating countries, as described in detail in the first ENCKEP Handbook.^11^ Below, we first present the main results from the questionnaires as viewed and prioritized during these workshops. The reflections and interpretations formed during these workshops are included in the Discussion and Conclusions.

## RESULTS

**FIGURE 1.**
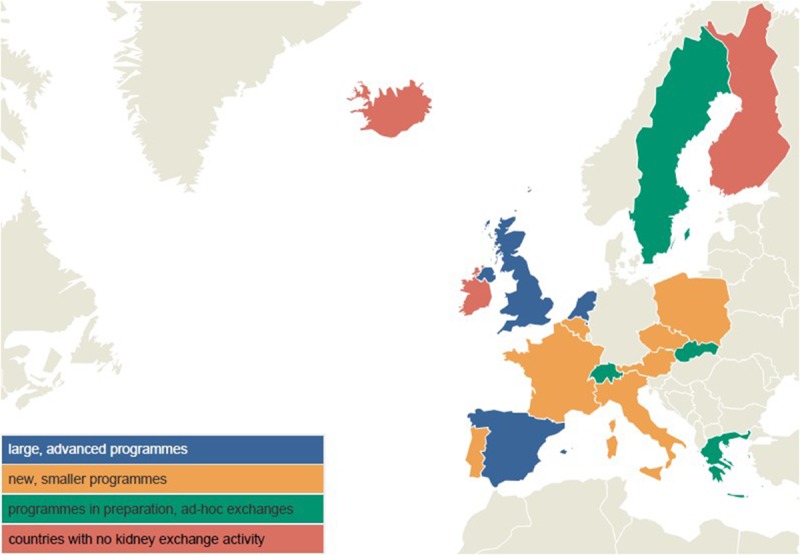
Development of KEPs by country.

In total, 17 of the 23 countries participating in ENCKEP completed the questionnaire. Countries were classified according to the level of development of their KEPs into 4 categories: (1) large, advanced programs, (2) smaller operational programs, (3) programs in preparation, and (4) countries without KEPs (Figure 1).

### Short Country Descriptions Based on the Responses from the Questionnaire

**The Netherlands** has the highest number of living kidney donations in Europe per million population (pmp), and was the first country in Europe to establish a nationally coordinated KEP in 2004. The program is coordinated by the Dutch Transplant Foundation in close collaboration with 8 transplant centers and a single Central Reference Laboratory for histocompatibility testing. From 2004 through 2016, the Dutch KEP has resulted in 284 transplants, facilitating exchanges up to 4 pairs.^12-23^ The **UK** Living Kidney Sharing Schemes includes all 4 nations (England, Wales, Scotland, Northern Ireland) and has been operational since 2007. It was made possible by the Human Tissue Acts 2004 (England, Northern Ireland, and Wales) and 2006 (Scotland). The program is administered by NHS Blood and Transplant and involves 23 transplant centers and 20 histocompatibility and immunogenetics (H&I) laboratories. It has become the largest operating KEP in Europe, with 250 recipient-donor pairs registered per matching run, and a total of 658 transplants reported.^24-26^ The **Spanish** national program was developed in 2009 by Organización Nacional de Transplantes involving 25 of the 39 transplant centers and 18 H&I laboratories in the country. The Spanish program is the second largest KEP in Europe after the United Kingdom with 110 donor-recipient pairs registered per matching run, and a total of 147 transplants reported.

As regard smaller programs, the *Austrian* KEP started in 2013 with 4 participating transplant centers, coordinated by the Medical University of Vienna, with immunological testing on-site.^27^ After conducting 11 transplants through 4 2-way exchanges and 1 3-way exchange between 2013 and 2016, Austria joined the Czech Republic to create 1 pool, and the first reported transnational exchange was performed in September 2016.^28^ The KEP in the **Czech Republic** started in 2011 and is organized by a single center, the Institute for Clinical and Experimental Medicine Prague. Despite the limited pool size, 60 transplants were performed.^29^ The reasons for this high number of transplants are: the flexibility in performing long exchanges nonsimultaneously (the Czech Republic has conducted 6- and 7-way exchanges), the fast laboratory testing that facilitates rematching in response to positive crossmatch tests, the involvement of altruistic donors and compatible pairs, and the possibility of performing ABOi and HLAi transplants after desensitization within the KEP. The **Polish** program started in 2015, organized by surgeons from the Department of General and Transplant Surgery, Medical University of Warsaw. Five transplants (through one 2-way and one 3-way exchange) had been performed in a single center (Warsaw) by 2016 and the first interhospital exchange was performed in 2017 between 2 centers.

The **Belgian** Living Donor Exchange Protocol was accepted in 2008 and the national KEP started in 2013, organized by the Kidney-Pancreas Committee and the Belgian Transplantation Society. The size of the pool is still limited, and 7 transplants have been reported through 2 2-way exchanges and 1 3-way exchange. The **French** KEP started in 2013, organized by the Agence de la Biomédecine. A total of 67 patients have been registered with a total of 10 transplants performed in 2-way exchanges. Altruistic donation is not legal in France, and exchanges involving more than 2 pairs are not yet allowed. The **Italian** KEP, run by the Italian National Transplant Centre, was established in 2006, but until 2014 only single-center exchanges were performed in the Pisa and Siena centers, achieving a total of 15 transplants. Since 2015 a further 16 transplants were conducted through long, but almost simultaneous, altruistic donor chains, involving up to 11 centers. The national KEP in **Portugal** started in 2010, administered by Instituto Português do Sangue e da Transplantação. Five transplant centers out of 8 and 3 H&I laboratories are participating. Fifty patients were registered and a total of 9 transplants were reported by the end of 2016.

In several countries the KEPs have just been created or are in preparation. **Sweden** started its national KEP in 2016. In 2017, the initiative was taken up by Scandiatransplant, under the name of ScandiaTransplant Kidney Exchange Program, involving **Sweden, Denmark**, and **Norway**. A matching policy has been agreed and 2-way exchanges are currently allowed.^30^ However, no transplants have been performed yet. In **Switzerland**, kidney exchange has been regulated by the Swiss Transplantation Law since 2007, but only a few ad hoc 2- and 3-way exchanges have been performed so far. In fact, in 1999, Europe’s first kidney exchange was carried out in Switzerland. However, there were legislative and organizational restrictions. Since these are solved, the Swiss KEP is expected to start in 2018.^31^ In **Greece**, a national KEP will be established by the Hellenic Transplant Organization (EOM) in the near future, involving 5 kidney transplant centers. There is no national KEP in **Slovakia** yet, but there have been 7 ad hoc 2-way exchanges between 2005 and 2015. Among those, 6 were performed in single centers (within 3 of the 4 transplant centers in Slovakia) and 1 exchange was between 2 centers.

We have no information on other operating KEPs in Europe. However, **Romania** had a single center KEP in Cluj-Napoca from 2001.^32,33^ Between 2001 and 2005, their medical team performed 56 transplants through 23 2-way exchanges, two 3-way exchanges and one 4-way exchange.

Regarding the countries that provided responses to our survey, but had no KEPs, **Finland** and **Iceland** are part of Scandiatransplant but they do not participate in ScandiaTransplant Kidney Exchange Program yet. Kidney exchange is not legally allowed in Finland; the Helsinki center performs all LDKT in the country. The LDKT program in Iceland is supported by a visiting surgeon from the United States, and patients travel to Sweden for a deceased donor kidney transplant (DDKT). Recipient-donor pairs of **Ireland** can register through a UK transplant center for entry into UK Living Kidney Sharing, altogether 6 transplants have been performed by that route.

### Statistics and Comparisons

**FIGURE 2.**
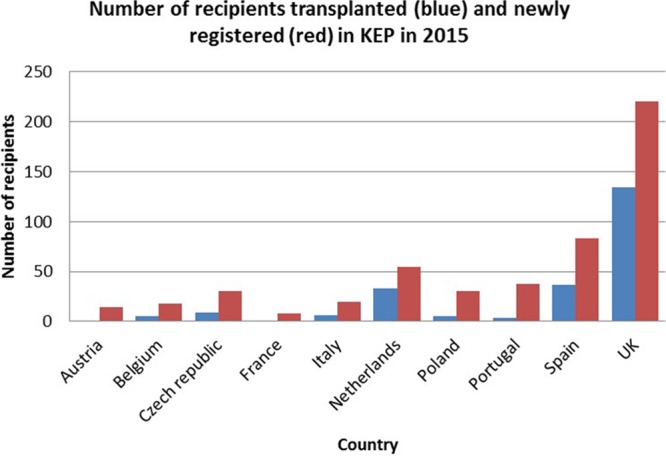
KEP activity: number of proceeding transplants and the number of new recipients registered in 2015 by country.

Regarding 12-month performance, the number of registrations and the number of transplantations conducted in 2015 can be seen in Figure 2.

The active KEPs in all 10 countries listed are organized centrally, although in the Czech Republic and Poland, the programs initially operated in the capitals, but they are now enlarging. Regarding legal restrictions, altruistic donation is not possible in France, Poland, Greece and Switzerland. In France and Portugal, only incompatible pairs can participate in the KEP. In France, only 2-way exchanges are possible. In principle, nonresident donor-recipient pairs can be involved in the majority of the KEPs except Portugal, where only resident recipients can join the KEP, and Belgium, where the recipient must be resident in a Eurotransplant country. Anonymity is a legal requirement in most countries and it is part of the protocol in all countries, except Poland. Compatible pairs can participate in KEPs in the advanced programs. In Spain, both pediatric recipients and compatible pairs are prioritized within the KEP. Usually organs travel rather than donors, except in the Netherlands, Slovakia and Switzerland, where the donors travel according to the protocol. Simultaneous surgeries for exchanges are required everywhere by protocol, except in the Czech Republic and Poland due to large exchanges and lack of operating theaters, respectively.

In most countries, there are national guidelines for donors and recipients to enter the KEP. All the donors and recipients are fully assessed before inclusion in a KEP and in some countries special rules apply for clinically complex donors. Usually large countries have multiple laboratories to carry out crossmatch testing and smaller countries use 1 laboratory only. In all of the KEPs the exchanges/chains are identified after a virtual crossmatch and then these are confirmed in a laboratory crossmatch. Most countries have nationally agreed definitions of HLA incompatibility and a commonly used mean fluorescence intensity threshold (between 2000 and 3000). Almost all countries consider results from historical samples as well, but the current ones are deemed most relevant. The practice regarding formal arrangements for reimbursing the expenses and loss of earnings of living donors varies between countries, but the countries with the most advanced KEPs have cost neutral reimbursement policies.

The algorithms which match recipients with donors mostly aim to maximize the number of transplants, in combination with a variety of other criteria. In many countries the optimization considers the HLA-match between donors and recipients and/or blood type compatibility. Blood type identical transplants can be prioritized. Additionally, algorithms might look for smaller donor-recipient age-differences and travel distances (coming from the same region). In the United Kingdom, donor-donor age difference is used as a tie-breaker between possible matches. The sensitization and the waiting time of the patient can also matter. Shorter exchange cycles are generally preferred in the solution.

We summarize these and other main features of the European KEPs in Table 1. We also included general data on the transplant activity of the countries covered in our survey in Table 2 and Table 3.

**TABLE 1.**
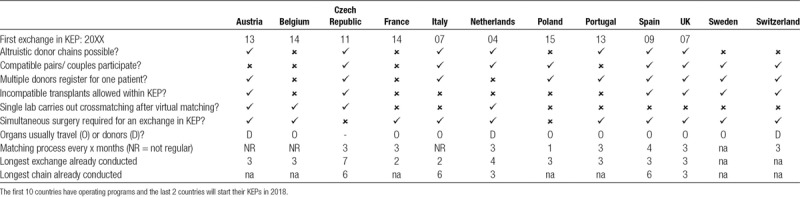
Summary of the characteristics of European kidney exchange programs

**TABLE 2.**
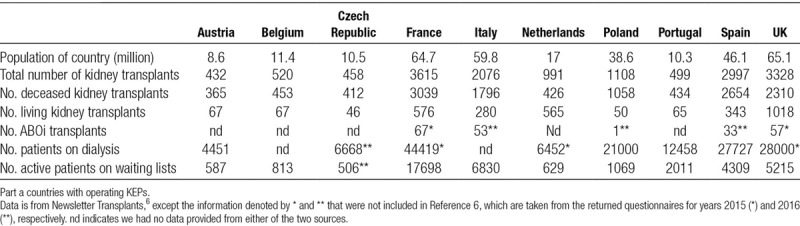
General information about the kidney transplantation activities of the 17 countries covered in the survey for 2016, Part A

**TABLE 3.**
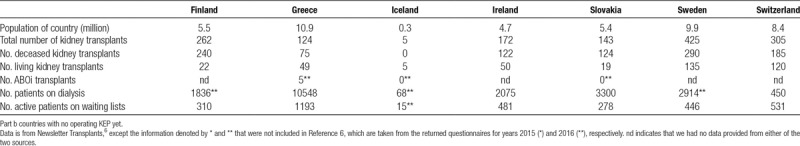
General information about the kidney transplantation activities of the 17 countries covered in the survey for 2016 from Newsletter Transplants, Part B

### Overview of Activity beyond Europe

There are several countries outside Europe where KEPs are operational, for example, in South Korea, United States, Canada, and Australia. For further information, we refer to 2 recent surveys^25,26^ on KEPs around the world. Here, we include a description of the situation in the United States, and information on one of their nationwide KEPs, ie, the United Network for Organ Sharing (UNOS), which was established in 2010. UNOS is a partner organization in the ENCKEP COST Action.

In the United States, UNOS (which administers the Organ Procurement and Transplantation Network [OPTN]), the National Kidney Registry and the Alliance for Paired Donation are the 3 KEPs operating nationwide. In addition, many regional and single-center programs exist within approximately 250 living donor transplant centers. Seventy centers are actively participating with UNOS from among the 160 centers registered with this organization. Of note is that approximately 200 centers have conducted at least 1 transplant in an exchange through an internal hospital program, a regional program, or one of the national programs in the United States. Some of the large transplant centers perform the majority of their exchanges in-house and report only their hard-to match patient-donor pairs to the national programs. One of the organizational implications of this fragmented system is that the national KEPs do not wait to build up their pools, as is common practice in Europe.

United Network for Organ Sharing conducts matching runs every week, and the National Kidney Registry and the Alliance for Paired Donation search for exchanges immediately after each registration. UNOS allows 2- and 3-way exchanges and 4-way chains for logistical reasons. Among the 173 transplants performed between 2010 and 2015, 52 were through 2-way exchanges 75 through 3-ways and 46 through altruistic chains. The high (90%) failure rate is the main issue in the US system. By the end of 2015, UNOS had 1628 registered patients and they identified 2246 matches in virtual crossmatch tests. Only 173 transplants proceeded, which is partly due to the practice of centers registering only hard-to-match patients and the lack of consistency in the HLA laboratory testing.

### Challenges and Opportunities

The survey identified the following main challenges for countries that do not yet operate a KEP: (1) lack of knowledge about KEPs; (2) lack of software tools; (3) lack of a legal framework; and (4) the small size of the country.

Descriptions of existing practices, as for instance provided above, as well as the ENCKEP COST Action itself, improve access to information and knowledge and serve to address the first challenge. Software to manage and optimize KEPs will also be investigated by ENCKEP, and future reports will enhance accessibility of existing technology for new countries.

The development of legislation to allow kidney exchanges (and its new modalities) is a task for policy makers and governments, in alignment with public opinion about living donor organ donation. Being well-informed about the operation and effects of existing KEPs across Europe may influence the beliefs and opinions of medical professionals, policy makers, the public and the government. Finally, as to the size of the country being a limitation, our results show that some medium-sized countries run effective KEPs or engage in international cooperation to this purpose.

Regarding the countries with operating KEPs, a key challenge reported by all countries is to maintain or increase the KEP pool size by encouraging new registrations. This holds particularly true for HLAi or ABOi pairs when routinely referred for desensitization treatment (as is for example the case in France, Italy, and Sweden). The accumulation of highly HLA sensitized recipients in the pool is an issue for several countries, making it difficult to find suitable matches for immunologically complex recipients. As an example, we note that in Spain the proportion of highly sensitized (PRA > 75%) patients has gradually increased from around 20% in 2010 to 50% in 2015.

There are essentially 3 categories of opportunities to improve the performance of the KEPs: (1) extending the national pools, (2) allowing new modalities in the exchange, and (3) increasing international cooperation.

#### (1) Extending National Pools

- Reaching full coverage in the national KEPs through the participation of all the transplant centers.
- Inclusion of compatible pairs to achieve better HLA or age match for the recipients involved and generating more exchange opportunities for other recipients.
- Inclusion of HLA incompatible pairs in preference to antibody removal, and inclusion of ABO incompatible pairs to avoid the costs and higher risks associated with desensitization programs (at least by registering them for a number of runs in the KEP before choosing these alternative treatments).
- Inclusion of altruistic (unspecified) donors to trigger KEP chains, where the transplants can be nonsimultaneous and in the form of long or “never ending” chains.
- Allowing multiple potential donors to register for 1 patient to improve the chances of identifying exchange cycles.

#### (2) Allowing New Modalities in the Exchange

- Extending the length of simultaneous exchange cycles and nonsimultaneous chains.
- Considering HLAi and ABOi transplantations for hyperimmunized patients, for them to receive a kidney that is immunologically better than his/her original donor’s, with the help of desensitization treatment.
- Improving the effectiveness of the selection of exchanges by fine-tuning the interaction between optimization and the virtual and laboratory crossmatch tests. Flexibility with finding alternative exchanges in the light of a positive crossmatch is important to maximize transplants.
- Establishing an optimal run frequency. Depending upon the dynamics of the pool, finding the right frequency may further enhance the performance of the KEP.

#### (3) Increasing International Cooperation

There are a number of different models for current international collaborations and plans: some are led by an advanced KEP, whereas other countries are joining together their small pools to create larger pools. In the United Kingdom, patient-donor pairs from Ireland are welcome to participate, and 6 recipients have already received transplants. Spain, with the second largest KEP in Europe, has started cooperation with Italy and Portugal. The Netherlands helped to establish the Belgian KEP by sharing their software. France and Switzerland signed a KEPs convention in 2015, but to date no run has succeeded in identifying matched pairs. France is also in negotiation with Belgium. Austria and the Czech Republic have already joined their pools and performed their first transnational exchange in September 2016,^28^ followed by a 3-way kidney exchange on December 5, 2017, involving 2 pairs in Prague and 1 in Vienna. Based on the Swedish initiative, Scandiatransplant has just started the coordination of a joint KEP involving Denmark, Norway, and Sweden. Eurotransplant is helping with the organization of the Belgian KEP and, theoretically, it is possible for a resident of a Eurotransplant country to join a KEP in another Eurotransplant country. International Collaboration is an opportunity that in turn poses new challenges. It requires many legislative, medical, financial, and ethical issues to be resolved. As the example of deceased organ sharing networks (such as Eurotransplant and Scandiatransplant) shows, such international cooperation is feasible and can be beneficial for all stakeholders, and especially the highly sensitized patients.

## DISCUSSION

This study provides a comprehensive review of the state of the art of European KEPs in LDKT, identifying their key characteristics and their main challenges. Our findings clearly reveal the complexity of the (interorganizational) structures and processes involved in KEPs. It appears that there is a considerable diversity among KEPs in participating countries, each having been developed in a different context and taking its own approach to address country-specific challenges. The most advanced programs with a longer history have experience of how to advance by continuous review and improvement. The emerging/developing programs can learn from these advancements as well as from other early stage programs to develop a model that is suitable for their requirements. Recognizing the differences in ethical viewpoints, legal frameworks, clinical practices, geography, population size, et cetera, it is clear that solutions that appear to work best in one country may be suboptimal in another and vice versa. One “model” does not fit all.

The same differences between systems imply that collaboration in the form of transnational KEPs adds new complexities regarding ethical, legal, clinical, and financial frameworks of the countries involved. New principles, conditions and objectives are called for to ensure that collaboration appropriately benefits all populations, recipients and donors involved. Such collaboration may particularly benefit the growing number of highly sensitized patients who are hard to match nationally.

### Recommendations for National KEPs

Based on our study we have identified the following key characteristics that define KEP effectiveness:

- a transparent, objective, and responsible donor selection system, including both physical and psychological screening;
- consistent and responsive systems and processes including matching software, immunological testing, organizational framework, and central coordination;
- clinical leadership to establish confidence in the KEP as the treatment of choice for immunologically complex recipients and compatible recipient-donor pairs seeking a better age or HLA match and to ensure that recipients and donors are appropriately informed about their treatment options;
- recipient and donor awareness to inform their decision-making and encourage participation in the KEP;
- a centralized follow up system, to monitor the effectiveness of the program in terms of the impact on the donor and recipient, and more generally in terms of overall outcomes;
- a culture of continuous improvement to develop the KEP in response to innovations in the field, clinician, and patient choice, and to actively manage potential risks (eg, diminishing pool size, nonproceeding transplants, low uptake of the KEP).

### Future Work

Based on the trends observed in the development of European KEPs, we expect that new national programs to be established and that countries with operating KEPs will advance their programs according to the opportunities described above. In particular, we believe that more countries will permit altruistic donors to trigger long chains as part of the proposed exchanges, more countries will allow the participation of compatible donors in the programs by providing quality guarantees for these patients, and desensitization treatment will be better coordinated with the KEPs.

Besides the improvement of the national programs, we expect the ongoing international collaborations to extend rapidly, partly facilitated by our ENCKEP COST Action, in particular for:

- sharing knowledge and expertise to support new, emerging, and developing programs;
- effective cross-border collaborations to improve pool size and optimization;
- advanced cross-border schemes to maximize options for immunologically complex recipients who remain unmatched within their own country KEPs.

Our ENCKEP network continues to conduct scientific research in an international and interdisciplinary collaboration on KEPs. The next steps are to identify best practices in modeling and optimization aspects of the KEPs, and to address the ethical and legal challenges of (inter)national KEPs.

As effective KEPs ultimately benefit all patients, we hope that the presented detailed description of current KEP practices will guide advances in practice, foster international cooperation, and promote access to transplantation for the growing population of ESRD patients.

## ACKNOWLEDGMENTS

The authors would like to thank the reviewers for their valuable comments which have helped to improve the presentation of this article.

## References

[1] Klarenbach S Barnieh L Gill J Is living kidney donation the answer to the economic problem of end-stage renal disease? Semin Nephrol. 2009;29:533 538. 19751899 10.1016/j.semnephrol.2009.06.010

[2] Smith CR Woodward RS Cohen DS Cadaveric versus living donor kidney transplantation: a Medicare payment analysis. Transplantation. 2000;69:311 314. 10670645 10.1097/00007890-200001270-00020

[3] McFarlane PA Should patients remain on intensive hemodialysis rather than choosing to receive a kidney transplant? Semin Dial. 2010;23:516 519. 21039877 10.1111/j.1525-139X.2010.00740.x

[4] U.S. Department of Health & Human Services. Organ Procurement and Transplantation Network Web site. https://optn.transplant.hrsa.gov/. Accessed April 18, 2018.

[5] GODT. Global Observatory on Donation and Transplantation Web site. http://www.transplant-observatory.org/. Accessed April 18, 2018.

[6] Newsletter Transplant; International figures on donation and transplantation 2016. EDQM. 2017;22.

[7] Glorie K Haase-Kromwijk B van de Klundert J Allocation and matching in kidney exchange programs. Transpl Int. 2014;27:333 343. 24112284 10.1111/tri.12202

[8] European Cooperation in Science and Technology. European Network for Collaboration on Kidney Exchange Programmes Web site. http://www.enckep-cost.eu/. Accessed April 18, 2018.

[9] European Cooperation in Science & Technology. COST Web site. http://www.cost.eu/about_cost/cost_member_states. Accessed April 18.

[10] Committee of Ministers, Council of Europe. European Committee on Organ Transplantation within the Council of Europe Web site. https://www.edqm.eu/sites/default/files/terms_of_reference_of_the_european_committee_on_organ_transplantation_2016-2017.pdf. Accessed April 18, 2018.

[11] Biró P Burnapp L Haase B Kidney Exchange Practices in Europe, First Handbook of the COST Action CA15210: European Network for Collaboration on Kidney Exchange Programmes (ENCKEP) 2017. Available from the authors upon request.

[12] de Klerk M Kal-van Gestel JA Haase-Kromwijk BJ Living Donor Kidney Exchange Program. Eight years of outcomes of the Dutch Living Donor Kidney Exchange Program. Clin Transpl. 2011 287 290. 22755421

[13] De Klerk M Van Der Deijl WM Witvliet MD The optimal chain length for kidney paired exchanges: an analysis of the Dutch program. Transpl Int. 2010;23:1120 1125. 20525019 10.1111/j.1432-2277.2010.01114.x

[14] Roodnat JI Zuidema W van de Wetering J Altruistic donor triggered domino-paired kidney donation for unsuccessful couples from the kidney-exchange program. Am J Transplant. 2010;10:821 827. 20199504 10.1111/j.1600-6143.2010.03034.x

[15] de Klerk M Zuidema WC IJzermans JN Alternatives for unsuccessful living donor kidney exchange pairs. Clin Transpl. 2010 327 332. 21696050

[16] de Klerk M Zuidema WC Ijzermans JN On chain lengths, domino-paired and unbalanced altruistic kidney donations. Clin Transpl. 2009 247 252. 20524290

[17] de Klerk M Witvliet MD Haase-Kromwijk BJ Hurdles, barriers, and successes of a national living donor kidney exchange program. Transplantation. 2008;86:1749 1753. 19104416 10.1097/TP.0b013e3181908f60

[18] de Klerk M Weimar W Ingredients for a successful living donor kidney exchange program. Transplantation. 2008;86:511 512. 18724217 10.1097/TP.0b013e318181fe3b

[19] de Klerk M Witvliet MD Haase-Kromwijk BJ A flexible national living donor kidney exchange program taking advantage of a central histocompatibility laboratory: the Dutch model. Clin Transpl. 2008 69 73. 19711512

[20] de Klerk M Witvliet MD Haase-Kromwijk BJ A highly efficient living donor kidney exchange program for both blood type and crossmatch incompatible donor-recipient combinations. Transplantation. 2006;82:1616 1620. 17198246 10.1097/01.tp.0000250906.66728.8d

[21] de Klerk M Haase-Kromwijk BJ Claas FH Living donor kidney exchange for both ABO-incompatible and crossmatch positive donor-recipient combinations. Transplant Proc. 2006;38:2793 2795. 17112831 10.1016/j.transproceed.2006.08.157

[22] Kranenburg LW Zuidema W Weimar W One donor, two transplants: willingness to participate in altruistically unbalanced exchange donation. Transpl Int. 2006;19:995 999. 17081229 10.1111/j.1432-2277.2006.00378.x

[23] de Klerk M Keizer KM Claas FH The Dutch national living donor kidney exchange program. Am J Transplant. 2005;5:2302 2305. 16095513 10.1111/j.1600-6143.2005.01024.x

[24] Johnson RJ Allen JE Fuggle SV Kidney Advisory Group, UK Transplant NHSBT. Early experience of paired living kidney donation in the United Kingdom. Transplantation. 2008;86:1672 1677. 19104403 10.1097/TP.0b013e3181901a3d

[25] Ferrari P Weimar W Johnson RJ Kidney paired donation: principles, protocols and programs. Nephrol Dial Transplant. 2015;30:1276 1285. 25294848 10.1093/ndt/gfu309

[26] Manlove D O’Malley G Paired and Altruistic Kidney Donation in the UK: algorithms and experimentation. ACM JEA. 2014;19: art. 2.6.

[27] Böhmig GA Fidler S Christiansen FT Transnational validation of the Australian algorithm for virtual crossmatch allocation in kidney paired donation. Hum Immunol. 2013;74:500 505. 23380140 10.1016/j.humimm.2013.01.029

[28] Böhmig GA Fronek J Slavcev A Czech-Austrian kidney paired donation: first European cross-border living donor kidney exchange. Transpl Int. 2017;30:638 639. 28236641 10.1111/tri.12945

[29] Fronek J Janousek L Marada T Paired Kidney Exchange program—is there potential for European cooperation? Single Czech institution experience with 26 paired transplants since 2011. Am J Transplant. 2014;98:614.

[30] Andersson T Kratz J Kidney exchange over the blood group barrier. Lund University Working Paper. 2016:11.

[31] Hadaya K Fehr T Rüsi B Kidney paired donation: a plea for a Swiss National Programme. Swiss Med Wkly. 2015;145:w14083. 25742633 10.4414/smw.2015.14083

[32] Lucan M Rotariu P Neculoiu D Kidney exchange program: a viable alternative in countries with low rate of cadaver harvesting. Transplant Proc. 2003;35:933 934. 12947805 10.1016/s0041-1345(03)00169-6

[33] Lucan M Five years of single-center experience with paired kidney exchange transplantation. Transplant Proc. 2007;39:1371 1375. 17580142 10.1016/j.transproceed.2007.02.081

